# Report and Discussion at the Royal Academy of Medicine, on Lithotomy and Lithotrity, &c.

**Published:** 1836-10

**Authors:** 


### Art. VIII.

*Rapport et Discussions à l'Académie Royale de Médecine sur la Taille et la Lithotritie, &c.*—8vo. *Paris,* 1835.

*Report and Discussion at the Royal Academy of Medicine, on Lithotomy and Lithotrity, &c.*

Until very recently, the provinces in this country were almost entirely deprived of scientific meetings for the purpose of discussing medical topics; and, in the metropolis and large towns, where such meetings are held, the most eminent persons, whether physicians or surgeons, seldom attend to take a part in the proceedings. It is otherwise in France, where a degree of natural vivacity and a strong disposition to be sociable conspire to bring gentlemen of our calling into strong collision in debate; whilst courtesy restrains the warmth of argument within the rules of moderation, and secures its being maintained in proper language.

Of the several medical societies of Paris, the "Royal Academy of Medicine" is the most attractive and the best supported; and, as often as any interesting subject is announced for discussion, the most renowned of the profession meet and take part in the proceedings, with all the weight of their years, experience, and eloquence. The pamphlet, of which the title is above prefixed, fully bears out these remarks: it contains the verbatim report of a discussion, prolonged through several sittings of the Academy, and arising out of a "Memoir upon Lithontripsy in Children," furnished by M. Leroy.

In this memoir the author has adduced five instances of the lithontriptic operation upon children under six years of age, in one of which a single sitting sufficed to effect a cure; the others required several trials, ending in the cure of two of the little patients, whilst one was left doubtful as to a portion of stone still remaining, and the fourth was freed from his disease by lithotomy.

MM. Sanson and Velpeau were appointed to *report* upon the memoir of M. Leroy, and they allowed him to have shewn the possibility of performing lithontripsy upon children at the tenderest age, without, however, proving its utility or its preference to lithotomy.

"This method of breaking down the stone deserves infinitely less commendation than is now generally accorded it; on the one hand, the dangers of lithotomy are represented in the strongest light, on the other the security of lithotrity is greatly exaggerated; and as often as an attempt is made to compare the two operations together, care is taken to avoid considering them under analogous circumstances."

Indulging in such remarks as these, the reporters upon the original memoir open the field for discussion. Hostilities once proclaimed, a regular assault and battery ensue, and, after much heavy cannonading on both sides, victory is awarded to neither, and Truth puts in a claim for more time and experience to settle finally the question in debate.

M. Amussat takes the lead in defence of the *new method.*

" Whence (he enquires) arises the aversion of some surgeons to lithotrity? in the first place, because it demands so much study and care; it is an entirely new art for them to learn; much practice, and a sort of love for the operation, are required for success; and it is moreover less brilliant than the old method by cutting. But the surgeon's aim should be to cure, not to make a display: for my own part I am never more satisfied than when I have either broken down a calculus which others would have removed by lithotomy, or reduced a hernia for which an operation had been proposed." (P. 17.)

This orator adds, that one great argument in favour of lithotrity is, that all medical men afflicted with stone in the bladder have of late years had recourse to it; whilst none have had reason to repent of their choice. M. Lisfranc confirms the justice of this remark by his own experience, and sums up a short speech by affirming that "lithotrity ought to be the general rule, and lithotomy the exception."

It is soon found out by M. Velpeau, that there is more assertion than proof in these speeches of the partisans of lithotrity: he calls for statistical details to argue upon, and (after receiving the assisting testimony of M. Sanson, that, "of the lithotomized in Paris, at least four out of five recover,") contrasts the two methods of operating in the following manner; the only difference in the translation we offer being the omission of some repetitions, and the correction of some small errors which we have found in the original text.

" 1. M. Civiale, according to a fourth statistical table of his operations, recently published, cured 18 out of 30 by lithotrity, 4 of the remaining 12 retaining a stone in the bladder, and 8 dying;—a brilliant result truly! and what would it be, if we were to examine the success of lithotrity in the provinces, where operators have not the dexterity of those in the metropolis?

" 2. Out of 1200 patients lithotomized at l'Hôtel Dieu and la Charité at Paris, there are reckoned 945 cures and 255 deaths. Saucerotte of Luneville obtained much more remarkable success, curing 1482 out of 1629. Dupuytren lost 61 out of 366. At the Leeds Hospital, of 197 operations, 28 proved fatal. Cheselden lost 24 out of 213 operated, and Frère Côme 19 out of 100. Under Souberbielle, 17 died out of 133. Dupuytren cut 70 patients by the bilateral method, and lost only 6. Crosse has given a very exact table of 704 operations for stone at the Norwich Hospital, of which 93 only were followed by death. Of 401 cases of lithotomy at Naples, in the last few years, 60 deaths are recorded. Pajola lost 5 in 50, Pansa 5 in 70, Ouvrard 5 in 60, Viricel 3 in 83, Martineau 2 in 83 in his own practice, and Dudley 1 in 72. Putting out of the question these last, which present results so extraordinarily fortunate, we find the following to be the proportion of fatal cases, viz. with Frère Côme, one of the most unsuccessful operators, one case in 5 fatal, with Souberbielle 1 in 6, Cheselden 1 in 9, Dupuytren by the bilateral method 1 in 12. Many other distinguished surgeons, who have not given an exact table of their operations, assert that they have obtained even greater success. Professor Smith, of America, estimates his losses as 1 in 18; Chelius in Germany as 1 in 22; Petrunti at Naples as 1 in 25 in private practice; and Santaro states that he has lost only one patient out of 56. It will surely be allowed that these statistical reports are worth more than those furnished by the lithotritors." (P. 23.)

M. Velpeau allows, notwithstanding, that lithotrity is a fortunate triumph of art over human infirmities, if kept within proper limits; but, when its too-ardent advocates represent it as being not more dangerous than passing a catheter, and as unattended by accidents, he puts some very puzzling questions.

"Do you reckon for nothing," (he says,) "those affections of the nervous system which prove fatal? or those sequels of an inflammatory kind, also proving fatal, as inflammation of the bladder, of the prostate gland, of the peritoneum, phlebitis, retention of urine, laceration of the urethra, and pains of almost every kind occasionally produced by this operation? The public are deluded by the promise that no pain shall be produced, whereas lithotrity in reality causes more pain than lithotomy. It is absolutely necessary that this extravagant enthusiasm should cool a little, and that lithotrity should be restrained within its proper and legitimate boundaries." (P. 25.)

The venerable Baron Larrey is present,—the same who braved Egyptian sands and Russian snows, and now finds the vicissitudes of an animated discussion very trying to his temperament: he censures the lithotritists, to whom he is opposed, for using some expressions not at all *academical*, and for alluding to the prejudices of antiquated members of the profession. We have the person of this veteran surgeon vividly depicted in our memory, and cannot refrain from expressing the wish that he may live long to dignify such meetings by his presence, and receive the respect due to him.

M. Roux states, in reference to his own experience, that one out of five or six adults die after lithotomy, and only one in twenty amongst children; but his calculations are given at a guess, and not based on any regular documents: he soon yields to M. Lisfranc in the debate, who again urges the superiority of lithotrity, upon the grounds of his own choice and experience; at the same time confessing that it cannot be exclusively employed.

With rejoinders from several of the orators already quoted, and some further remarks by M. Sanson, not of sufficient importance to claim a place here, the discussion for the first evening closes.

At the next meeting, M. Souberbielle is the only speaker from whom any remarks of value escape: of 133 patients lithotomized by him, no less than thirty had previously submitted to a trial of lithotrity; he denies altogether the harmlessness of this method, and the perfection to which it is pretended the operation has been brought as to mechanism.

"The same accidents, which occurred at first, still happen daily, and in the hands of the same surgeons; thus M. Turgot in 1824 found the urethra and the rectum perforated; in 1826 he met with laceration of the former passage, and urinary abscesses; in 1828 M. Le Sénécal found a perforation of the urethra and of the corpus cavernosum; M. Gasselin, a laceration of the same canal and an abscess in the anterior wall of the bladder; the urethra was torn, and urine infiltrated in the case of a certain general; and in the preceding year, in the case of M. Hector Chaussier, the bladder was twice seized, and portions of its mucous membrane brought away by the stone-breaking instrument!" (P. 39.)

Being ourselves no partisans of either method, we are glad to give to our readers, as amply as space and time will allow, the different arguments contained in the little book we have taken up, for their instruction and amusement.

On the third evening of the discussion, M. Lisfranc endeavoured to reduce to a more just estimate the statistical deductions of M. Velpeau in favour of lithotomy; showing that, when females are taken from the list, and children distinguished from adults, the average success in males of the latter class is, in France, one in three or four, and in England (though more satisfactory, according to the published accounts of Smith and Crosse, at the different hospitals of Bristol, Leeds, Norwich,) it never amounts to more than one in six or seven.

"Hence it is evident, (says M. Lisfranc,) that neither is lithotrity so fatal, nor lithotomy so fortunate, as has been asserted; and it may moreover be observed, that, in proportion as lithotrity shall become popular, calculous patients, deterred hitherto through fear of the cutting operation, will more easily seek the advice of the surgeon: thus there will be fewer patients suffering from calculi of large size and of many years' standing; and as it is acknowledged that lithotrity is preferable for small calculi, it will in time become the general method." (P. 49.)

The question is brought to its right bearing by some judicious remarks of M. Amussat, who represents the two methods of operating as not directly opposed to each other, each having its special indications. "We have not to determine generally whether one operation be better than the other, but what cases we should select for the one or the other method." Dividing the calculous cases into two classes, he claims for lithotrity more than his opponents could well spare,—"all cases where the bladder is healthy and the concretion small," even though there be two or three calculi; and also where the calculus is as big as a nut, if soft and friable; leaving to the lithotomist all patients with large calculi, particularly such as are of a firm consistence, or friable, or filling the bladder, or complicated with vesical catarrh. We suspect it will be some time before both parties become reconciled to these conditions, convinced as we feel ourselves, by much actual observation, that lithotomy can prove uniformly or generally successful only in cases where the bladder is tolerably healthy and the calculus of moderate size. We confess our great surprise to find any practised operator proclaiming the superiority of lithotomy, by its being applicable to all cases of stone: it can, we grant, be performed in almost all cases, but is it advisable? We know not how it is in France, but in this country a majority of those adults who are affected with stone in the bladder die without being operated on, chiefly from apprehension of the pain or danger attending lithotomy; and, if the operation be indiscriminately performed, the mortality becomes so great as to deter those who ought to undergo it. The road to the lithotritist's success is cleverly marked out by M. Amussat: let the lithotomist exercise as much caution, or ere long he will be seldom called upon, except to undertake the cure of patients in whom lithotrity has been tried and proved ineffectual.

The operation of lithotomy can scarcely undergo any future very considerable changes for the better, as regards the plan and mechanism: lithotrity (a triumphant discovery, it must be confessed,) is in its infancy, and, although greatly over-rated, and falling short in practice in the hands of most surgeons, save those whose attention is almost exclusively devoted to it, may go on improving till it arrive at a degree of success equal to what its warm supporters would persuade us to believe it has already attained, but which is not shown by authentic documents, even in the countries where it is most cultivated, France and Great Britain.

But we return to the discussion, which was adjourned to a fourth sitting, with an increased number of visitors.

M. Lepelletier repeated the doctrine of M. Roux, that "lithotomy is applicable to all calculous patients, without exception: lithotrity, on the contrary, is suited only to a certain proportion, and under favorable conditions." He regards lithotomy as the easier operation, and thinks it no small recommendation of this or any other operation, that it can be well done by most surgeons. The reproach of lithotrity is, that few can perform it with sufficient dexterity to command success; and it is urged, that it ought not to be undertaken where the calculus is situated partly in the urethra or in the ureter, or when adherent to the bladder, or lodged partly in a sac formed by the inner coat of this organ; nor in cases where the calculi are numerous, complicated with disease of the kidneys, of the prostate glands, or of the urethra; nor in cases where the irritability of the patient renders the different manœuvres for breaking up the stone impracticable. According to our ideas, most of these conditions, as where the kidneys, prostate gland, or urethra are diseased, forbid equally the performance of either operation. The preference of lithotrity must rest upon different arguments from these. In looking prospectively, we cannot be so rash as to limit the degree of dexterity which may be attained, or the perfection of mechanism that may be arrived at: it seems sufficient to state, which we feel bound to do, that the greatest tact and experience are required in the new operation, and that the proportion of patients will increase as surgeons become better qualified to operate on them. We have always felt respect for M. Civiale, on account of the candour he has shown in placing the difficulties of stone-grinding and stone-crushing before the public. There is equal reason to be satisfied with M. Amussat, who uses this strong argument, evincing the improvement of the art:

"At first, when the operation was performed by successive perforations of the stone, I found, amongst the patients who applied to me, about an equal proportion suited to each method; but since the great improvements in the instruments," (it might also very justly be added, the different plan of performing the operation, the *lithontriptic* method of M. Heurteloup,) "I operate by breaking down the calculus in at least two thirds of my patients, and practise lithotomy more and more rarely." (P. 117.)

We have thus endeavoured to give a portion of the arguments on both sides: the reader who desires more must peruse the original treatise, which is not less interesting as a specimen of free and able discussion, than as tending to settle the value of one of the best improvements in modern surgery. In conclusion, we will give a part of the eloquent *impromptu* of M. Velpeau, with which the meeting of the Academy closes; a speech breathing the excess of liberalism, yet showing more the native talent than the cool and correct judgment of the speaker.

"Where consists the cruelty of drawing a rigorous comparison between lithotomy and lithotrity? where lies the danger of lithotomy? *in the wound.* And what bad consequences may the inflicting this wound give rise to? it may give rise: 1, to hemorrhage, which is rarely serious; 2, to a wound of the rectum, which is rare and of little importance; 3, to a perforation of the bladder, still more rare; 4, to accidents affecting the nervous system, also very rare; 5, to cystitis, often mortal; 6, to peritonitis, phlebitis, or urinary infiltrations, which are not less formidable; 7, to fistulæ, incontinence, impotence, which may be considered as simple infirmities, from which the patient most commonly recovers; lastly some fragments may remain in the bladder! Here is a chapter of accidents long enough, no doubt; but wait a moment, and you shall see that the lithotritist travels over a path covered with thorns. The dangers of lithotrity result from the necessity of keeping a large and straight instrument in the urethra. It will be answered, perhaps, that the instrument now used is curved; but there is a great mistake on this point. The curvature, being situated at the extremity of the instrument, renders its introduction more easy; but the portion which occupies the urethra is nevertheless quite straight; hence arises compression and violence to the prostate gland, the membranous portion of the urethra, and about the symphysis of the pubis; hence shocks to the nervous system, to such a degree as to prove fatal, contusions, lacerations, infiltrations; also abscess of the urethra, of the prostate gland, of the scrotum or perinæum; hence sufferings so severe that, although inadvertently designated trifling by M. Amussat, they were aptly compared by one of his patients to the agony attending the extraction of a tooth, the most frightful perhaps that can be borne. But this is not all; severe attacks of fever, dangerous inflammations of the joints, urethritis, phlebitis, hemorrhage, perforation of the bladder or rectum, fistulæ, are also consequences of this *instrumentation*, without taking into the account that cystitis, and inflammation of the ureters and kidneys are more common after lithotrity than after lithotomy. We may further add, retention of urine, peritonitis, instruments broken and which cannot be removed, the suffering and mischief caused by calculous fragments lodging in the urethra, and we shall then have an idea of the *little danger* attending *lithotrity*, *that mild and gentle operation!* The public are misled, because the accidents of lithotomy occur either immediately, or so soon after the operation, as not to be separated from it; whilst those of lithotrity arise more gradually, even at a period so remote that both operator and patient believe them foreign to the treatment. Again, lithotomy either kills or cures; but lithotrity often does neither, at the same time that it lays the foundation for insidious mischief, which shews itself in disease so gradually that, without any breach of faith, it is assigned to some other cause. In short, this new operation, which is to a certain extent good, has hitherto been treated as a spoiled child, its advocates concealing all its defects, and setting forth, with too much ostentation, its good qualities: if we let this state of things go on, one abuse will follow another, until, under the support of this kind of nepotism the operation will be ruined, since it is admitted that nepotism answers no better in the end for a surgical operation, than for the success of an individual. The language employed in its favour threatens to throw this operation into the hands of mechanics and adventurers; the means of rescue are, to remove it from the bosom of its parents and family, and to merge it in the circle of general surgery, where, being viewed by broad daylight, the evils which attend it will be separated from the good, and the operation will in due time be estimated at its just value."

Since the foregoing notice was sent to the press, we have received Dr. Civiale's work, entitled "Parallèle des divers Moyens de Traiter les Calculeux," which contains a more complete and elaborate review of the arguments in favour of each of the rival methods. We shall give, in an early Number, an account of this valuable treatise, which, we believe, will shortly appear in an English dress from the pen of our own celebrated lithotritist, Mr. Costello.

